# HspBP1 in Complex with the Peptide of the Innate Immunity Protein Tag7 is Able to Lyse Tumor Cells Carrying TNFR1 Receptor

**DOI:** 10.1134/S1607672923700631

**Published:** 2024-01-08

**Authors:** E. A. Romanova, D. M. Yurkina, D. V. Yashin, L. P. Sashchenko, G. P. Georgiev

**Affiliations:** Institute of Gene Biology, Russian Academy of Sciences, Moscow, Russia

**Keywords:** Tag7, HspBP1, tumor cells, cytotoxicity, 17.1 peptide

## Abstract

The search for new cytotoxic agents capable of lysing tumor cells is an important task in the fight against cancer. Here we have shown that the HspBP1 protein, the chaperone of the heat shock protein Hsp70, is able to form a complex with the previously discovered peptide (17.1) of the innate immunity protein Tag7. Experiments using thermophoresis demonstrated that the affinity of the Tag7 protein peptide 17.1 to the HspBP1 molecule is 100 times higher than that of the full-sized Tag7 molecule. The addition of the 17.1–HspBP1 complex to tumor cells induces apoptosis and necroptosis in them. The results obtained in this work can be used to develop promising antitumor drugs.

Understanding the mechanisms of tumor cell death and identifying new compounds that induce this death are very important for the development of new strategies for antitumor immunotherapy.

Among the first cytotoxic proteins, the tumor necroptosis factor (TNF) was described. TNF is now known to be a cytokine and plays a key role in the immune response [1]. The functional activity of TNF is realized through interaction with receptors on the cell surface and induction of an intracellular signal [2]. TNF receptors are well characterized and intensively studied [3]. The most well-known specific TNF receptor is TNFR1 [4]. TNFR1 is present in many tissues on various cells, including tumor cells [5]. This receptor induces two alternative processes of programmed cell death in cells: apoptosis and necroptosis. Apoptosis involves triggering a cascade of specific proteases, caspases, whose activation ultimately leads to the cleavage of DNA to nucleosomes [6]. When caspase 8 is blocked, genetically or due to external stimuli, activation of RIP1 and RIP3 phosphokinases and activation of the necroptosis program are observed [7].

Expanding the range of ligands for this receptor will facilitate the creation of new ones through immunotherapy. We have previously shown that the Tag7 protein, also known as (PGRPLY1), the gene for which was discovered in mice at our Institute [8], is a ligand for the TNFR1 receptor [9]. By binding to the receptor, it can block its interaction with other ligands, including TNF. In combination with Hsp70, Tag7 induces alternative cytotoxic processes in tumor cells [10].

We also described the HspBP1 protein, which is involved in the regulation of the interaction of the Tag7–Hsp70 complex with cells [11]. It is a co-chaperone that regulates the ATPase activity of Hsp70 [12]. The proteins can bind to Tag7 and inhibit the cytotoxic activity of the Tag7–Hsp70 complex. We found that each of these proteins competes with Hsp70 for the Tag7-binding site with the formation of an inactive complex. These results suggest that they bind to the same Tag7 fragment as Hsp70. Relatively recently, we identified a peptide fragment of Tag7 (peptide 17.1), which is responsible for binding to Hsp70 and is necessary for the manifestation of cytotoxic activity. We created the 17.1–Hsp70 complex, which also has a cytotoxic effect on tumor cells. Our preliminary data suggest that Mts1 can also form a cytotoxic 17.1–Mts1 complex. Here, we investigated whether HspBP1 can interact with this peptide and form a cytotoxic complex.

The purposes of this work were (1) comparative characterization of complexes of the Tag7 protein and its functional peptide fragment 17.1 with the HspBP1 protein and (2) study of the cytotoxic activity of the Tag7–HspBP1 and 17.1–HspBP1 complexes.

L929 cells (mouse fibroblasts) were cultured in DMEM medium (PanEco, RA) supplemented with 10% fetal bovine serum (HyClone, United States). The culture was obtained from the bank of cell lines of the Blokhin Russian Cancer Research Center of the Russian Academy of Medical Sciences. To measure cytotoxicity, proteins and protein complexes were added to the L929 culture at a concentration of 1 nM, unless a different concentration was specified, and incubated for 3 or 20 h. Cytotoxicity was measured using a Cytotox kit (Promega, United States) according to the manufacturer’s method. To inhibit cytotoxicity, blocking antibodies to TNFR1 were used at a concentration of 10 μg/mL (Invitrogen, United States). Data from 5 different experiments are used.

Recombinant HspBP1 protein was obtained as described earlier [11]. A column with CNBr-activated Sepharose 4B (GE Healthcare, Chicago, IL, United States) conjugated to 17.1 was prepared according to the manufacturer’s protocol. HspBP1 protein was loaded onto this column, and the column was washed with PBS containing 0.5 M NaCl and PBS only. The protein was eluted with 0.25 M triethylamine (TEA) at pH 12. The eluted material was resolved by SDS-PAGE and blotted onto a nitrocellulose membrane. We used polyclonal rabbit antibodies to HspBP1 or Tag7 (Abcam, Cambridge, United Kingdom; 1 : 15000; 1 h). HRP-conjugated anti-rabbit antibodies (Abcam, Cambridge, United Kingdom; 1 : 15 000; 1 h) were used for detection. Results were visualized using the ECL Plus kit (GE Healthcare, Chicago, IL, United States) according to the manufacturer’s protocol. Chemiluminescence was recorded with an iBright instrument (Thermo Fisher Scientific, Boston, MA, United States).

Purified HspBP1 was fluorescently labeled using Alexa Fluor 633 (Eugene, OR, United States) according to the manufacturer’s instructions. HspBP1 (200 nM) was incubated for 20 min with each compound in the dark at room temperature at 16 different concentrations obtained by serial dilution starting with the highest soluble concentration. Samples were loaded into glass capillaries (Monolith NT Capillaries) and analyzed by thermophoresis using a Monolith NT 115 nanotemperature instrument (IR laser power 10%). The signal quality was monitored with a NanoTemper Monolith instrument to detect possible ligand autofluorescence, precipitation, aggregation, or ligand-induced changes in photobleaching rate. Experiments were performed in triplicate and processed using affinity analysis software (MO Control v.1.6.1, Nano-Temper).

Data were analyzed using Statistica 6.1 software (StatSoft®). The Shapiro–Wilk test was used to confirm the normality of data distribution. Results are presented as mean ± SD. Statistically significant differences were determined using the *t* test. A value *p* < 0.05 was considered statistically significant.

We previously showed that HspBP1 interacts with Tag7 immobilized on CN-Br Sepharose, indicating the formation of a Tag7–HspBP1 complex. L929 cells were used to measure cytotoxic activity, since they bear the TNFR1 receptor and are highly sensitive to both TNF and the Tag7–Hsp70 complex. Here, we confirmed that the addition of HspBP1 inhibits the cytotoxic activity of the Tag7–Hsp70 complex in a dose-dependent manner. The results presented in Fig. 1a show a drastic decrease in the cytotoxicity of the Tag7–Hsp70 complex with increasing concentration of the inhibitory protein HspBP1. The Tag7–HspBP1 complex does not have cytotoxic activity; however, the addition of Hsp70 caused an increase in cytotoxic activity with increasing Hsp70 concentration. Maximum cytotoxicity was reached at a 5-fold excess of Hsp70 relative to the Tag7–HspBP1 complex (Fig. 1b). These results suggest that Hsp70 and HspBP1 compete for the same Tag7-binding site.

**Fig. 1.**
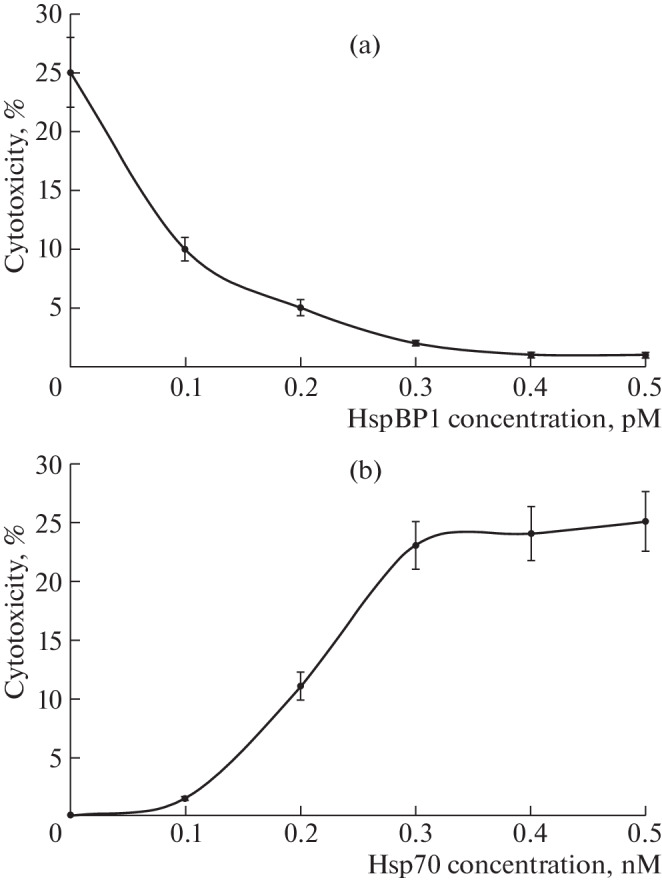
(a) The addition of an increasing concentration of HspBP1 to the Tag7–Hsp70 complex inhibits its cytotoxic activity. (b) The addition of an increasing concentration of Hsp70 to the Tag7–HspBP1 complex leads to the appearance of cytotoxic activity.

Previously, we also showed that Hsp70 binds to a 12-membered peptide fragment located in the C-terminal region of the Tag7 polypeptide chain (peptide 17.1). Here, we studied the interaction of peptide 17.1 with HspBP1 using affinity chromatography. The HspBP1 sample was applied to CN-Br Sepharose with immobilized peptide 17.1.

The bound material was analyzed by SDS-PAGE followed by Western blot with specific antibodies against HspBP1 (Fig. 2). It can be seen that the eluate contains a protein with a molecular weight of 40 kDa, which interacts with specific antibodies to HspBP1. Therefore, the formation of a 17.1–HspBP1 complex is possible.

**Fig. 2.**
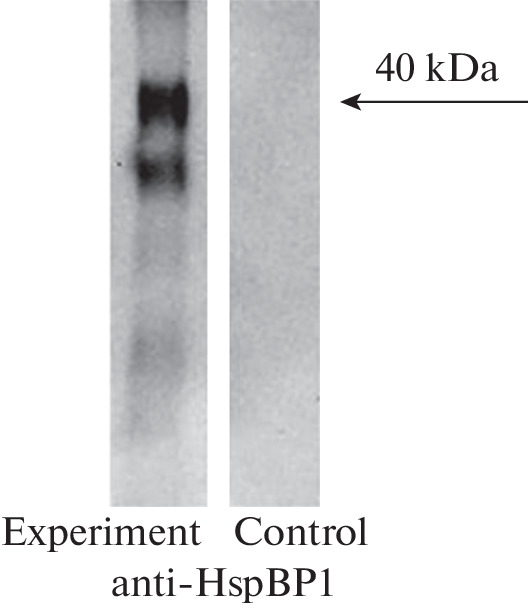
HspBP1 addition to 17.1-Sepharose (experiment) or to pure Sepharose (control), resolved by SDS–PAGE in 12% polyacrylamide gel and stained using antibodies to HspBP1.

Next, a comparative assessment of the affinity of these complexes was performed. We used quantitative microscale thermophoresis to evaluate the protein–protein interaction of HspBP1 with Tag7 and peptide 17.1. When Tag7 and its peptide were added to labeled HspBP1, clear binding curves with increasing concentrations of the studied components were obtained (Figs. 3a, 3b). Thermophoresis data show that ${{K}_{D}}$ for the Tag7–HspBP1 complex is 328 nM, which indicates a low affinity of these compounds and low stability of the complex. The interaction of the Tag7 fragment, peptide 17.1, with HspBP1 can be considered highly specific, since ${{K}_{D}}$ is 3.91 nM. It can be assumed that such a complex has a higher stability.

**Fig. 3.**
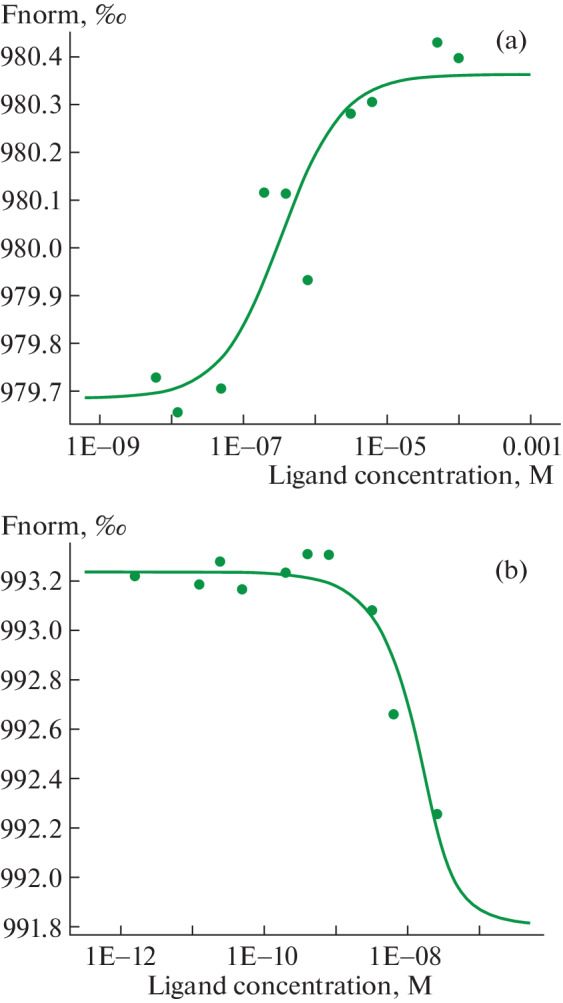
Thermophoretic dissociation curves for the interaction of Tag7–HspBP1 (a) and 17.1–HspBP1 (b).

The next stage of the study was to evaluate the cytotoxic activity of the obtained complexes. It was previously shown that Tag7–Hsp70 and 17.1–Hsp70 complexes are cytotoxic [13]. As mentioned above, Tag7 is involved in the induction of cytotoxicity by interacting with the TNFR1 receptor, which induces alternative cytotoxic processes, apoptosis and necroptosis. It is also known that maximum apoptotic cytotoxicity is reached after 3 h of interaction of the ligand with the receptor, necroptosis develops after 20 h of such interaction. In view of this, cytotoxic activity was determined after 3 and 20 h of interaction of the ligand with cells. The results obtained showed the absence of cytotoxic activity.

Four complexes (Tag7–HspBP1, as well as 17.1–HspBP1 with 1 : 1 and 1 : 2 stoichiometry) were prepared and added to tumor cells. Cytotoxic activity was determined after 3 and 20 h of incubation (Figs. 4a, 4b).

**Fig. 4.**
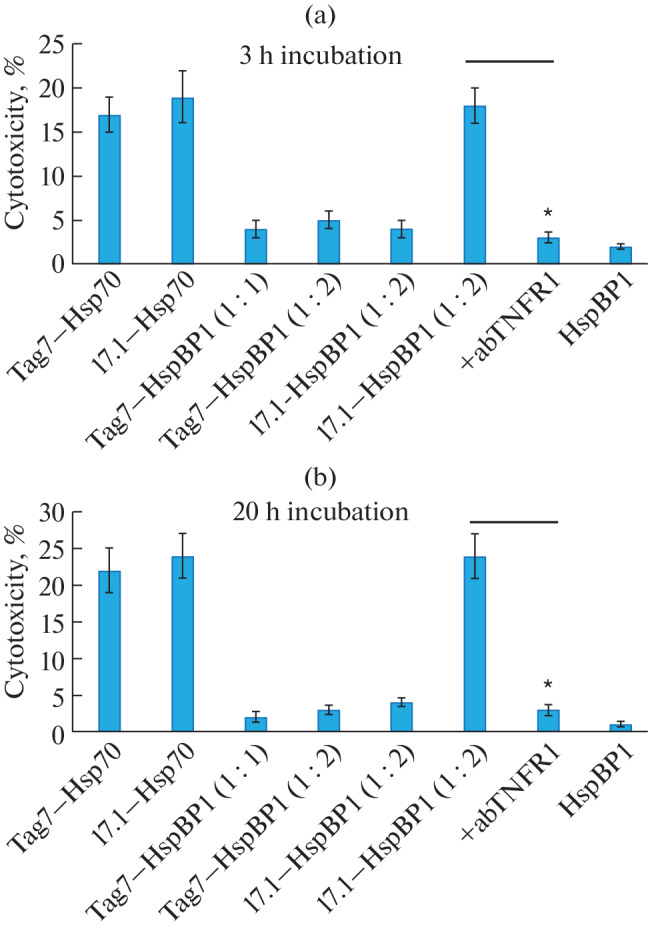
Cytotoxic activity of complexes after (a) 3 and (b) 20 h of incubation with L929 tumor cells. * *p* < 0.05.

It can be seen that the Tag7–HspBP1 complex did not have cytotoxic activity even when the amount of the second component increased. The 17.1–HspBP1 1 : 1 complex also had no cytotoxic activity. However, the 17.1–HspBP1 1 : 2 complex exhibited cytotoxicity after 3 and 20 h of incubation with cells, which was suppressed by the addition of anti-TNFR1 antibodies. These results suggest that the cytotoxic 17.1–HspBP1 complex induces apoptotic and necroptotic processes of programmed cell death in tumor cells when the complex interacts with the TNFR1 receptor.

We previously showed that peptide 17.1 has high affinity for Hsp70, ${{K}_{D}}$ amounted to 7.16 nM and was comparable to for ${{K}_{D}}$ Hsp70 with full-length Tag7 (1.73 nM) (13). ${{K}_{D}}$ For 17.1–HspBP1, equal to 3.91 nM is also close to these values. All three complexes had cytotoxic activity. for the complex ${{K}_{D}}$ HspBP1 with full-length Tag7 is significantly higher and was 328 ntM, which indicates low activity and weak stability of the complex. It can be assumed that the lack of cytotoxic activity inTag7–HspBP1 is associated with its dissociation upon interaction with cells. Thus, a correlation between the affinity of protein–protein interaction and cytotoxic activity has been established.

It is currently unclear why the full–length Tag7 has a low affinity for HspBP1. Possibly, a structural fragment of this protein prevents the strong binding of HspBP1 to the Tag7 polypeptide chain region corresponding to peptide 17.1. This issue can be finally resolved after X-ray diffraction analysis. Previously, in our studies, we showed that HspBP1 is an inhibitor of the cytotoxic activity of the Tag7–Hsp70 complex. Here, we demonstrated that, in the presence of the Tag7 peptide, to which it binds much more strongly, it can participate in the induction of cytotoxic activity.

To summarize, it can be noted that a new high-affinity cytotoxic complex has been obtained that induces the death of tumor cells when it interacts with the TNFR1 receptor.

## References

[1] Carswell E.A. (1975). An endotoxin-induced serum factor that causes necroptosis of tumors. Proc. Natl. Acad. Sci. U. S. A..

[2] Siegmund D., Wajant H. (2023). TNF and TNF receptors as therapeutic targets for rheumatic diseases and beyond. Nat. Rev. Rheumatol.

[3] Huyghe J., Priem D., Bertrand M.J.M. (2023). Cell death checkpoints in the TNF pathway. Trends Immunol.

[4] Shi G., Hu Y. (2023). TNFR1 and TNFR2, which link NF-κB activation, drive lung cancer progression, cell dedifferentiation, and metastasis. Cancers.

[5] Gough P., Myles I.A. (2020). Tumor necroptosis factor receptors: pleiotropic signaling complexes and their differential effects. Front. Immunol.

[6] Ashkenazi A., Salvesen G. (2014). Regulated cell death: signaling and mechanism. Annu. Rev. Cell Dev. Biol.

[7] Roberts J.Z., Crawford N., Longley D.B. (2022). The role of ubiquitination in apoptosis and necroptosis. Cell Death Differ.

[8] Kustikova O.S. (1996). Cloning of the *tag7* gene expressed in metastatic mouse tumors. Genetika.

[9] Yashin D.V. (2015). Tag7 (PGLYRP1) in complex with Hsp70 induces alternative cytotoxic processes in tumor cells via TNFR1 receptor. J. Biol. Chem..

[10] Yashin D.V. (2016). The Tag7-Hsp70 cytotoxic complex induces tumor cell necroptosis via permeabilization of lysosomes and mitochondria. Biochimie.

[11] Yashin D.V. (2011). The heat shock-binding protein (HspBP1) protects cells against the cytotoxic action of the Tag7-Hsp70 complex. J. Biol. Chem..

[12] Bracher A., Verghese J. (2023). Nucleotide exchange factors for Hsp70 molecular chaperones: GrpE, Hsp110/Grp170, HspBP1/Sil1, and BAG domain proteins. Subcell Biochem.

[13] Yurkina D.M. (2023). Short peptides of innate immunity protein Tag7 (PGLYRP1) selectively induce inhibition or activation of tumor cell death via TNF receptor. Int. J. Mol. Sci.

